# Desensitization of delayed-type hypersensitivity in mice: suppressive environment

**DOI:** 10.1155/S0962935193000274

**Published:** 1993

**Authors:** Takashi Katsura, Kazuo Kobayashi, Michio Hosaka, Sachiko Sugihara, Tsuyoshi Kasama, Keita Kasahara, Stanley Cohen, Takeshi Yoshida

**Affiliations:** 1 The First Department of Internal Medicine Showa University School of Medicine 1-5-8 Hatanodai Shinagawa-ku Tokyo 142 Japan; 2 Department of PathoLogy Hahnemann University Philadelphia USA; 3 Tokyo Institute for Immunopharmacology Tokyo Japan

## Abstract

The systemic injection of high doses of antigen into a preimmunized animal results in transient unresponsiveness of cell-mediated immune responses. This phenomenon is known as desensitization. Serum interleukin 2 (IL-2) activity was found transiently in desensitized mice at 3 h after the antigen challenge. These mice could not reveal antigen nonspecific delayed-type hypersensitivity (DTH) 1 d after the challenge. Specific suppression of DTH was observed at later stages. Sera from 3 h desensitized mice showed suppressive effects on DTH in preo immunized mice. Administration of recombinant IL-2 into preimmunized mice led to the failure of development of DTH to antigens. These observations suggest that IL-2 plays an important role in the suppressive environment.


Mediators of Inflammation, 2, 205–210 (1993)

**THE systemic injection of high doses of antigen into a preimmunized animal results in transient unresponsiveness of cell-mediated immune responses. This phenomenon is known as desensitization. Serum interleukin 2 (IL-2) activity was found transiently in desensitized mice at 3 h after the antigen challenge. These mice could not reveal antigen nonspecific delayed-type hypersensitivity (DTH) 1 d after the challenge. Specific suppression of DTH was observed at later stages. Sera from 3 h desensitized mice showed suppressive effects on DTH in preimmunized mice. Administration of recombinant IL-2 into preimmunized mice led to the failure of development of DTH to antigens. These observations suggest that IL-2 plays an important role in the suppressive environment.**

**Key words:** Delayed-type hypersensitivity, Desensitization, Immunosuppression, Interleukin 2

### Desensitization of delayed-type hypersensitivity in mice: suppressive environment

**Takashi Katsura,[1] Kazuo Kobayashi,[1,CA] Michio Hosaka,[1] Sachiko Sugihara,[1] Tsuyoshi Kasama,[1] Keita Kasahara,[1] Stanley Cohen,[2] and Takeshi Yoshida[3]**

[1]The First Department of Internal Medicine, Showa University School of Medicine, 1-5-8 Hatanodai, Shinagawa-ku, Tokyo 142, Japan; [2]Department of Pathology, Hahnemann University, Philadelphia, USA; [3]Tokyo Institute for Immunopharmacology, Tokyo, Japan

[CA] Corresponding Author

### Introduction

Desensitization of delayed-type hypersensitivity (DTH) refers to the transient loss of reactivity to antigen(s) observed in preimmunized animals following systemic administration of a large amount of antigen.[1–5] When animals sensitized with two or more antigens are desensitized by the intravenous injection of one of the immunizing antigens without adjuvant, DTH to this antigen is reduced or ablated (specific desensitization).[1,2] In addition, there is a marked reduction in skin reactivity after challenge with other antigen(s) to which the animals have been immunized, but not desensitized (nonspecific desensitization).[3–5] Previous studies have shown that desensitization is a multistage process,[5–7] involving an initial overpopulation of lymphokines such as migration inhibition factor, followed by inhibition of lymphokine production.

The present study was conducted to explore the suppressive environment in desensitized mice by using passive transfer of sera or lymph node cells. Furthermore, the restoration of the immunosuppression by the administration of exogenous IL-2 and/or by an IL-2 dependent antigen-reactive T lymphocyte clone mediating *in vivo* DTH has been attempted.

### Materials and Methods

*Immunization/desensitization:* Methylated bovine serum albumin (MBSA) was purchased from Sigma, St Louis, MO. Purified protein derivative (PPD) was purchased from Connaught Laboratories, Willowdale, Ontario, Canada. Female BALB/c mice (Charles River Japan, Tokyo, 6–10 weeks of age) were immunized with 125 μg MBSA emulsified in complete Freund's adjuvant (Difco, Detroit, MI) containing 2 mg/ml of heat-killed *Mycobacterium tuberculosis*.[8–10] Ten days after immunization, mice were desensitized by injection with a total of 1.5 mg MBSA (0.5 mg i.v. and 1 mg i.p.) as described previously.[11]

*Cutaneous DTH responses:* Mice were challenged with 40 μg/0.02 ml antigen (MBSA or PPD) into the hind footpad.[8,10] The difference of footpad thickness at 24 h after and just before challenge injection was measured. Measurements were made in triplicate for each mouse tested by using an engineer's micrometer (Starret, Athol, MA), and the mean swelling (mm) ± S.E.M. was calculated.[8,10]

*Transfer of lymph node cells and MBSA-reactive cloned T cells:* Draining lymph nodes were removed from donor mice and single cell suspensions were prepared.[8,10] The cells showed 90–95% viability as assessed by trypan blue dye exclusion. Recipient mice were given $5 \times 10^7$ cells/0.5 ml intravenously. An IL-2 dependent MBSA-reactive T lymphocyte clone from BALB/c mice immunized with MBSA has been established.[12] The cells are capable of mediating *in vivo* MBSA-induced DTH response in naive syngeneic mice in the presence of both MBSA

© 1993 Rapid Communications of Oxford Ltd and IL-2. Surface markers of the cells were $CD4^+$. In brief, MBSA-reactive T-cell suspensions ($1 \times 10^5$ cells/0.02 ml) were injected into the hind footpads of desensitized mice together with IL-2 (4 U) and MBSA (40 μg). Recipient mice were challenged with antigens for DTH within 1 h of the transfer. The swelling was measured 24 h after the challenge.

*Serum transfer:* Recipient mice were injected with 0.5 ml of serum intravenously. The treatment was given on the tenth or thirteenth day after immunization, and then recipients were tested for DTH.

*IL-2:* IL-2 activity was measured by a standard IL-2 assay[13] using cytotoxic T-lymphocyte line (CTLL) (kindly supplied from Dr K. A. Smith, Department of Medicine, Dartmouth Medical School, Hanover, NH), and the activity was expressed as half-maximal units/ml. Recombinant human IL-2 (specific activity, $1 \times 10^7$ U/mg, a gift from Shionogi Pharmaceutical Co., Osaka, Japan) was injected subcutaneously into mice. Footpads were challenged with MBSA or PPD within 1 h following the injection and 24 h swelling was then measured.

*Statistical analysis:* Analysis of variance (ANOVA) was used to determine the statistical significance between experimental and control groups; $p$ values less than 0.05 were considered significant.

### Results

*Failure of antigen-induced footpad responses in desensitized mice:* As shown in Fig. 1, a significant lack of footpad responses elicited by either MBSA (78–80% suppression) or PPD (83–89% suppression) was seen in desensitized mice at 1 d after desensitization when compared to immunized mice. Subsequently, abrogation of MBSA (59% suppression) but not PPD (7% suppression) induced responses was found in desensitized mice from day 3 through day 7. Thus, desensitization of cutaneous DTH is composed of antigen nonspecific and specific stages.

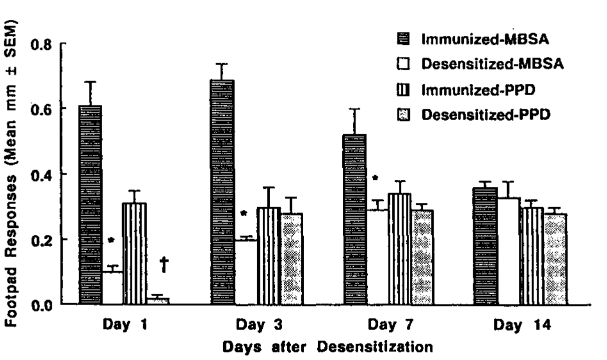

FIG. 1. Suppression of footpad responses in desensitized mice. Immunized mice were challenged with MBSA (immunized-MBSA) and PPD (immunized-PPD). Desensitized mice were challenged similarly. Significant suppression, $^*p < 0.02$, $^\dagger p < 0.01$.

*Adoptive transfer of lymph node cells:* As shown in Table 1, recipient immunized mice given lymph node cells either from 3 or 24 h desensitized mice showed control levels of footpad swelling. In contrast, MBSA (36% inhibition) but not PPD induced footpad responses were suppressed in recipient mice injected with cells of 72 h desensitized mice. No suppression of MBSA and PPD induced DTH was observed when lymph node cells from 0 h

Table 1. Adoptive lymph node cell transfer between desensitized and immunized mice[a]

| Donor | | Recipient | Footpad swelling (mm ± S.E.M.) in recipient mice | |
|---|---|---|---|---|
| Desensitized | Immunized | | MBSA | PDD |
| 3 h | – | Immunized | 0.54 ± 0.08 (0%)[b] | 0.24 ± 0.03 (12%) |
| – | 3 h | Immunized | 0.55 ± 0.06 (2%) | 0.29 ± 0.02 (−7%) |
| 24 h | – | Immunized | 0.51 ± 0.04 (6%) | 0.26 ± 0.01 (4%) |
| – | 24 h | Desensitized | 0.13 ± 0.02[c] (76%) | 0.09 ± 0.02[c] (67%) |
| – | – | Immunized | 0.54 ± 0.03 | 0.27 ± 0.03 |
| – | – | Desensitized | 0.10 ± 0.04[c] (81%) | 0.05 ± 0.03[c] (81%) |
| 72 h | – | Immunized | 0.36 ± 0.06[c] (36%) | 0.26 ± 0.01 (−8%) |
| – | 72 h | Desensitized | 0.29 ± 0.01[c] (49%) | 0.24 ± 0.02 (0%) |
| – | – | Immunized | 0.57 ± 0.04 | 0.24 ± 0.03 |
| – | – | Desensitized | 0.24 ± 0.04[c] (58%) | 0.25 ± 0.03 (−4%) |

[a] Lymph node cells ($5 \times 10^7$ cells/0.5 ml) from desensitized or immunized mice were transferred into immunized or desensitized mice. Footpads of these mice were challenged with antigens within 1 h after the transfer and the 24 h footpad swelling was then determined. Data represent the mean swelling (mm) ± S.E.M. from three separate experiments of three mice per each condition.
[b] Numbers in parentheses indicate percent suppression of footpad swelling as compared to those of control immunized mice.
[c] Significant suppression ($p < 0.01$) was observed as compared to footpad swelling of control immunized mice.

**Table 2.** Antigen nonspecific suppression of DTH by endogenous IL-2 containing serum injected into immunized mice[a]

| Serum from mice | Time (h) after desensitization | Footpad response (mean mm ± S.E.M.) in recipient mice | |
|---|---|---|---|
| | | MBSA | PPD |
| Desensitized | 3 | 0.35 ± 0.04[b] (42%)[c] | 0.20 ± 0.05[b] (44%) |
| Immunized | 3 | 0.65 ± 0.04 (−8%) | 0.36 ± 0.01 (0%) |
| Nonimmunized | 3 | 0.59 ± 0.03 (2%) | 0.38 ± 0.05 (−6%) |
| Desensitized | 24 | 0.54 ± 0.03 (10%) | 0.33 ± 0.04 (8%) |
| Immunized | 24 | 0.56 ± 0.06 (7%) | 0.32 ± 0.05 (12%) |
| Nonimmunized | 24 | 0.62 ± 0.10 (−3%) | 0.35 ± 0.03 (3%) |
| Desensitized | 72 | 0.35 ± 0.01[b] (42%) | 0.34 ± 0.05 (6%) |
| Immunized | 72 | 0.62 ± 0.06 (−3%) | 0.35 ± 0.06 (3%) |
| Nonimmunized | 72 | 0.63 ± 0.08 (−5%) | 0.37 ± 0.04 (−3%) |
| Normal mice | – | 0.55 ± 0.03 (9%) | 0.37 ± 0.08 (−3%) |
| None | – | 0.60 ± 0.09 | 0.36 ± 0.05 |

[a] Sera were injected intravenously into recipient immunized mice (0.5 ml i.v./recipient mouse). Serum 3 h desensitized mice contained endogenous IL-2 activity (50–65 U/ml). As controls, serum obtained from immunized mice injected with saline was used, nonimmunized mice injected with MBSA (1.5 mg/mouse), or normal BALB/c mice. Such control sera showed no IL-2 activity. Data represent the mean footpad swelling (mm) ± S.E.M. from three separate experiments of four mice per each condition. Serum pooled from 10 to 20 mice was used in each experiment.

[b] Significant suppression ($p < 0.01$) when compared to immunized mice without injection of serum.

[c] Values in parentheses indicate percent suppression of footpad swelling when compared to controls.

desensitized mice were transferred into immunized mice (data not shown). Lymph node cells from immunized mice were capable of transferring DTH into nonimmunized mice. Alternatively, no effect was found by cell transfer from desensitized or immunized mice to desensitized mice.

*Transfer of suppression of DTH by sera:* As shown in Table 2, serum from 3 h desensitized mice was capable of inhibiting DTH induced by either MBSA or PPD (42–44%) in immunized mice. The suppression was not due to residual antigen in donor mouse serum since the suppression was antigen nonspecific and 3 h serum from nonimmunized mice injected with MBSA showed no inhibition. Sera of 3 h desensitized mice contained endogenous IL-2 (Fig. 2). The activity was induced in MBSA preimmunized mice challenged with MBSA but not with unrelated antigen (egg albumin and keyhole limpet haemocyanin). Immunization procedures themselves could not induce the IL-2 activity since the serum from sensitized mice challenged with saline showed no IL-2 activity. Furthermore, MBSA itself had no effects on release of serum IL-2 activity because the serum from nonimmunized mice injected with MBSA did not contain IL-2 activity. Similar results were obtained by using mice immunized with other antigens and desensitized with the respective antigen. Thus, administration of a large amount of antigen into immunized mice could induce serum IL-2 transiently. In contrast, serum from naive, immunized and 24 h desensitized mice had no effects on DTH immunized mice.

*Inhibition of cutaneous DTH by* in vivo *administration of exogenous IL-2:* As shown in Table 3, preimmunized mice treated with recombinant IL-2 exhibited antigen nonspecific suppression of footpad responses induced by either MBSA or PPD in a dose-dependent fashion. Suppression of DTH was observed when mice were injected with ≥50 U of IL-2 per mouse but not with 10 U. To confirm the suppressive effects of excess IL-2 on DTH,

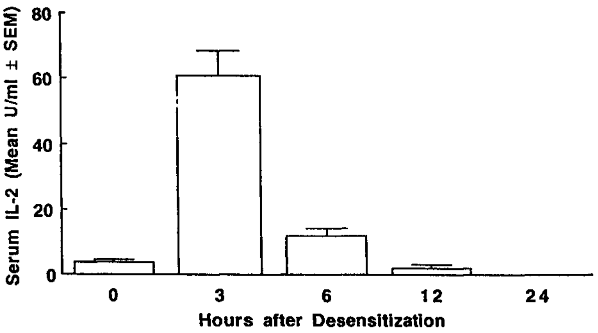

FIG. 2. Serum IL-2 activity in desensitized mice. Because CTLL could respond to IL-4 and IL-2, anti-IL-2 receptor (Boehringer Mannheim Biochemica, Indianapolis, IN) and anti-murine IL-4 monoclonal antibodies (TexStar, Dallas, TX) were used to confirm IL-2. The serum activity of 3 h desensitized mice was inhibited by anti-IL-2 receptor antibody but not by anti-IL-4 antibody. No detectable IL-2 activity was found in sera of immunized mice injected with saline and naive mice challenged with MBSA.

**Table 3.** Failure to restore the impaired footpad response in desensitized mice by exogenous IL-2[a]

| Mice | Units | Swelling (mean mm ± S.E.M.) | |
|---|---|---|---|
| | | MBSA | PPD |
| Immunized | 500 | 0.23 ± 0.06 (63%)[b] | 0.14 ± 0.04 (55%)[c] |
| | 100 | 0.34 ± 0.08 (46%)[c] | 0.18 ± 0.05 (45%)[c] |
| | 50 | 0.36 ± 0.05 (43%)[c] | 0.20 ± 0.06 (33%)[c] |
| | 10 | 0.51 ± 0.07 (19%) | 0.26 ± 0.08 (21%) |
| | 0 | 0.63 ± 0.08 | 0.31 ± 0.06 |
| Desensitized 3 h | 500 | 0.12 ± 0.07 (82%)[b] | 0.02 ± 0.08 (94%)[b] |
| | 100 | 0.10 ± 0.09 (85%)[b] | 0.03 ± 0.07 (91%)[b] |
| | 50 | 0.14 ± 0.10 (79%)[b] | 0.05 ± 0.08 (85%)[b] |
| | 10 | 0.13 ± 0.09 (81%)[b] | 0.03 ± 0.09 (91%)[b] |
| | 0 | 0.10 ± 0.06 (85%)[b] | 0.04 ± 0.03 (91%)[b] |
| Desensitized 72 h | 500 | 0.26 ± 0.07 (62%)[c] | 0.06 ± 0.08 (82%)[b] |
| | 100 | 0.27 ± 0.09 (60%)[c] | 0.08 ± 0.07 (76%)[b] |
| | 50 | 0.30 ± 0.10 (55%)[c] | 0.05 ± 0.08 (85%)[b] |
| | 10 | 0.30 ± 0.09 (55%)[c] | 0.10 ± 0.09 (70%)[b] |
| | 0 | 0.28 ± 0.02 (58%)[c] | 0.29 ± 0.01 (12%) |

[a] Data represent the mean mm ± S.E.M. from three different experiments of three mice per each condition. Values in parentheses indicate percent suppression of footpad response.
[b] Significant suppression, $p < 0.01$.
[c] Significant suppression, $p < 0.02$.

recombinant IL-2 was injected into desensitized mice. As shown in Table 3, administration of recombinant IL-2 was unable to restore the suppression of both antigen specific (MBSA) and nonspecific (PPD) DTH in desensitized mice.

*Incapability of transferring DTH in desensitized mice by the cloned MBSA reactive T cells:* As shown in Table 4, the cell clone was incapable of transferring MBSA induced DTH in desensitized mice at both early and later stages of desensitization.

### Discussion

In this study, it has been demonstrated that there are two stages of desensitization. These include antigen nonspecific desensitization at an early stage (day 1) and antigen specific desensitization at a later stage (days 3–7). Also, it has been shown that serum IL-2 activity transiently appeared in the mice 3 h after desensitization, and that, in a subsequent step, these mice were unable to express T-cell-mediated immune responses such as DTH.

**Table 4.** *In vivo* administration of MBSA-reactive T-lymphocytes mediating DTH is unable to induce antigen-specific footpad swelling in desensitized mice[a]

| Time after desensitization | Injection of T-cells[b] | Footpad swelling (mean mm ± S.E.M.) | |
|---|---|---|---|
| | | MBSA | PPD |
| 0 h | | | |
| Desensitized | + | 0.09 ± 0.01 (82%)[c] | 0.07 ± 0.02 (79%) |
| Desensitized | – | 0.06 ± 0.03 (88%) | 0.05 ± 0.01 (85%) |
| Immunized[d] | + | 0.50 ± 0.07 (0%) | 0.32 ± 0.05 (6%) |
| Immunized | – | 0.50 ± 0.02 | 0.34 ± 0.04 |
| 72 h | | | |
| Desensitized | + | 0.24 ± 0.04 (51%) | 0.29 ± 0.05 (9%) |
| Desensitized | – | 0.29 ± 0.02 (41%) | 0.31 ± 0.06 (3%) |
| Immunized | + | 0.47 ± 0.02 (4%) | 0.33 ± 0.07 (0%) |
| Immunized | – | 0.49 ± 0.02 | 0.32 ± 0.05 |

[a] The MBSA-reactive T-lymphocyte clone ($1 \times 10^5$ cells/0.02 ml) was injected into the footpads of desensitized mice along with MBSA (40 μg/0.02 ml) and IL-2 (4 U/0.02 ml). Data represent the mean swelling (mm) ± S.E.M. from three separate experiments of three mice per each condition. Similar results were obtained from 24 h desensitized mice injected with the cloned T cells.
[b] The T-cells could induce footpad swelling response to MBSA in nonimmunized mice in the presence of IL-2 and MBSA, 0.24 ± 0.03 mm. The cells were unable to induce PPD-induced footpad swelling in nonimmunized mice, 0.08 ± 0.02 mm.
[c] Values in parentheses show percentage suppression of footpad swelling as compared to control immunized mice.

Administration of recombinant IL-2 into pre-immunized mice induced antigen nonspecific suppression of DTH. In addition, neither exogenous IL-2 nor cloned antigen reactive T-cells could restore DTH in desensitized mice. These results suggest that IL-2-dependent regulatory mechanisms are responsible for the immunosuppression in desensitized mice.

The exact mechanisms of desensitization of DTH still remain unknown. There are several possibilities to explain the suppressive mechanisms by induction of suppressor cells,[14] antigen-mediated interference with effector cell function,[2,15,16] and compartmentalization of effector cells.[17] The nature of passive transfer of desensitization states by serum or immunologically competent cells remains controversial. Previous studies on antigen nonspecific desensitization of immunized guinea-pigs suggest that the suppression of DTH is due to several lymphokine-dependent mechanisms.[5–7,18] In fact, exogenous administration of lymphokines such as migration inhibition factor into immunized animals was capable of inducing the passive antigen nonspecific desensitization.[5–7,18] Antigen nonspecific DTH was inhibited by administration of sera containing endogenous IL-2 and recombinant IL-2 (Tables 2 and 3). Also, suppressed DTH in desensitized mice was unable to be restored by recombinant IL-2 (Table 3). Administration of exogenous IL-2 into cancer patients showed inhibition of DTH.[19,20] Taken together, the suppression of such mice may be mediated by lymphokine-dependent suppression mechanisms. Therefore, it was shown that the suppressed DTH could not be restored by administration of IL-2 and/or an IL-2 dependent antigen-reactive T cells.

It has been reported that exposure of cloned helper T-lymphocytes to IL-2 can induce the antigen unresponsive state, and that unresponsive helper T-lymphocytes are markedly impaired in their ability to produce IL-2 after antigenic restimulation.[21] This may support the present results that loss of helper cell activity is responsible for antigen nonspecific desensitization by excess IL-2 in sera. These two suppressive components including lymphokine-dependent mechanisms and loss of helper cells observed in desensitized mice at the early stage (nonspecific desensitization) may be closely related. Because type 1 helper T-lymphocytes producing IL-2 are responsible for classical cell-mediated $CD4^+$ functions such as DTH,[22,23] it is possible to assume that the interaction between soluble mediators and cells leads to the suppressive environment in the early stage of desensitization.

By contrast, antigen specific desensitization of DTH may be caused by antigen specific suppressor cells in lymph nodes of desensitized mice at later stage. Suppressor cells may be able to generate suppressor factor(s) into the serum, because the experiment of serum transfer from 72 h desensitized mice to immunized mice could inhibit specific DTH response to desensitized antigen (MBSA) but not immunized antigen (PPD). Again, neither exogenous IL-2 nor antigen reactive cloned T-cells could restore antigen specific DTH in desensitized mice at this stage. In the early stage of desensitization, a large amount of circulating serum IL-2 was found. It is known that IL-2 can induce suppressor cells.[24,25] It has been suggested that exposure of helper T-cells to exogenous IL-2 induces unresponsiveness to antigenic restimulation[26] and inhibits IL-2 production.[21] These findings may suggest that antigen nonspecific desensitization (early stage) is a prerequisite for the induction of antigen specific desensitization (later stage). The mechanisms of desensitization of cutaneous DTH may be composed of complicated immunological events such as an IL-2/lymphokine dependent regulatory system and loss of helper cell function. A soluble lymphocyte mediator, presumably IL-2, may play a central role in the development of the suppressive environment found in desensitized mice.

The desensitization of DTH mimics the state of clinical anergy seen in various human granulomatous[27] and lymphoproliferative diseases.[28] Interestingly, it is possible to demonstrate circulating lymphokines in the face of cutaneous unresponsiveness in human patients[27,28] and experimental animals[5,8,10,29] with these conditions. As mentioned above, this model is useful for investigating regulation of DTH as well as the role of IL-2 *in vivo*.

### References

1. Uhr JW, Papenheimer AM. Delayed hypersensitivity. III. Specific desensitization of guinea pigs sensitized to protein antigens. *J Exp Med* 1958; **108:** 891–904.
2. Claman HN, Jaffe BD. Desensitization of contact allergy to DNFB in mice. I. Description of a model system. *J Immunol* 1983; **131:** 2682–2686.
3. Dwyer JM, Kantor FS. *In vivo* suppression of delayed hypersensitivity: prolongation of desensitization in guinea pigs. *J Exp Med* 1975; **142:** 588–599.
4. Sonozaki H, Papermaster V, Yoshida T, Cohen S. Desensitization: effects on cutaneous and peritoneal manifestations of delayed hypersensitivity in relation to lymphokine production. *J Immunol* 1975; **115:** 1657–1661.
5. Yoshida T, Cohen S. Biological control of lymphokine function. *Fed Proc* 1982; **41:** 2480–2483.
6. Kobayashi K, Suko M, Yoshida T, Cohen S. Antigen-nonspecific and specific suppression of lymphokine production in desensitized guinea pigs. *Cell Immunol* 1986; **97:** 325–334.
7. Suko M, Yoshida T, Cohen S. Desensitization V: suppression of MIF production by lymphokine-activated macrophages. *Cell Immunol* 1985; **96:** 49–60.
8. Kobayashi K, Allred C, Castriotta R, Yoshida T. Strain variation of *Bacillus Calmette–Guerin*-induced pulmonary granuloma formation is correlated with anergy and the local production of migration inhibition factor and interleukin-1. *Am J Pathol* 1985; **119:** 223–235.
9. Kobayashi K, Allred C, Cohen S, Yoshida T. Role of interleukin 1 in experimental pulmonary granuloma in mice. *J Immunol* 1985; **134:** 358–364.
10. Kobayashi K, Allred C, Yoshida T. Mechanisms of suppressed cell-mediated immunity and impaired antigen-induced interleukin 2 production in granuloma-bearing mice. *J Immunol* 1985; **135:** 2996–3003.
11. Cone RE, Gerardi DA, Davidoff J, Petty J, Kobayashi K, Cohen S. Quantitation of T-cell antigen-binding molecules (TABM) in the sera of nonimmunized, immunized, and desensitized mice. *J Immunol* 1987; **138:** 234–239.

12. Kobayashi K, Cohen S, Yoshida T. Characterization of the antigen-reactive T-cell line mediating *in vivo* delayed type hypersensitivity established by antigen-induced interleukin 2. *J Exp Pathol* 1989; **4:** 163–179.
13. Gillis S, Ferm MM, Ou W, Smith KA. T-cell growth factor: parameters of production and a quantitative microassay for activity. *J Immunol* 1978; **120:** 2027–2032.
14. Swamy PK, Dwyer JM, Kantor FS. Desensitization in mice: T-cell requirement for nonspecific suppression of delayed-type hypersensitivity. *Cell Immunol* 1981; **60:** 308–313.
15. Polak L, Turk JL. Studies on the effect of systemic administration of sensitizer in guinea pigs with contact sensitivity to organic metal compounds. I. The induction of immunological unresponsiveness in already sensitized animals. *Clin Exp Immunol* 1968; **3:** 245–251.
16. Polak L, Gleick H. Differing mechanisms of tolerance and desensitization to DNCB in guinea pigs. *Eur J Immunol* 1975: **5:** 94–99.
17. Schlossman SF, Levin HA, Rocklin RE, David JR. The compartmentalization of antigen-reactive lymphocytes in desensitized guinea pigs. *J Exp Med* 1971; **134:** 741–750.
18. Papermaster V, Yoshida T, Cohen S. Desensitization. II. Passive transfer of the desensitized state by serum from desensitized animals. *Cell Immunol* 1978; **35:** 378–391.
19. Atkins MB, Gould JA, Allergretta M, *et al.* Phase I evaluation of recombinant interleukin-2 in patients with advanced malignant disease. *J Clin Oncol* 1986; **4:** 1380–1391.
20. Kolitz JE, Welte K, Wong GY, *et al.* Expansion of activated T-lymphocytes in patients treated with recombinant interleukin 2. *J Biol Response Modifiers* 1987; **6:** 412–429.
21. Wilde DB, Prystowsky MB, Ely JM, Vogel SN, Dialynas DP, Fitch FW. Antigen-reactive cloned helper T-cells. II. Exposure of murine cloned helper T-cells to IL-2-containing supernatant induces unresponsiveness to antigenic restimulation and inhibits lymphokine production after antigenic stimulation. *J Immunol* 1984; **133:** 636–641.
22. Mosmann TR, Coffman RL. TH1 and TH2 cells: different patterns of lymphokine secretion lead to different functional properties. *Ann Rev Immunol* 1989; **7:** 145–173.
23. Janeway CA Jr., Carding S, Jones B, *et al.* $CD4^+$ T-cells: specificity and function. *Immunol Rev* 1988; **101:** 39–80.
24. Nakauchi H, Ohno I, Kim M, Okumura K, Tada T. Establishment and functional analysis of a cloned, antigen-specific suppressor effector T-cell line. *J Immunol* 1984; **132:** 88–94.
25. Ting C-C, Yang SS, Hargrove ME. Induction of supressor T-cells by interleukin 2. *J Immunol* 1984; **133:** 261–266.
26. Wilde DB, Fitch FW. Antigen-reactive cloned helper T-cells. I. Unresponsiveness to antigenic restimulation develops after stimulation of cloned helper T-cells. *J Immunol* 1984; **132:** 1632–1638.
27. Yoshida T, Siltzbach LE, Masih N, Cohen S. Serum-migration inhibitory activity in patients with sarcoidosis. *Clin Immunol Immunopathol* 1979; **13:** 39–46.
28. Cohen S, Fisher B, Yoshida T, Bettigole RE. Serum migration-inhibition activity in patients with lymphoproliferative diseases. *N Engl J Med* 1974; **290:** 882–886.
29. Yoshida T, Cohen S. Lymphokine activity *in vivo* in relation to circulating monocyte levels and delayed skin reactivity. *J Immunol* 1974; **112:** 1540–1547.

ACKNOWLEDGEMENTS. This work was supported by grants from National Institutes of Health (HL-23982 and AI-16706), Ministry of Education, Science and Culture of Japan, Showa Medical Foundation, Mochida Memorial Foundation for Medical and Pharmaceutical Research, Tokyo, Japan.

**Received 19 January 1993; accepted in revised form 10 March 1993**
